# A Qualitative Study on Primary Care Integration into an Asian Immigrant-specific Behavioural Health Setting in the United States

**DOI:** 10.5334/ijic.3719

**Published:** 2018-07-03

**Authors:** Kris Pui Kwan Ma, Anne Saw

**Affiliations:** 1 Department of Psychology, DePaul University, 2219 N. Kenmore Ave, Chicago, US

**Keywords:** integrated care, behavioural health, primary care, health disparities, ethnic minorities

## Abstract

**Introduction:**

Integrating primary care and behavioural health services improves access to services and health outcomes among individuals with serious mental illness. Integrated care is particularly promising for racial and ethnic minority individuals given higher rates of chronic illnesses and poorer access to and quality of care compared to Whites. However, little is known about integrated care implementation in non-White populations. The aim of this study is to identify facilitators and barriers to successful implementation of primary care-behavioural health integration in a multilingual behavioural healthcare setting.

**Methods:**

Seven focus groups and five semi-structured interviews were conducted with 41 patients and 5 providers participating in integrated care in a community mental health clinic in California serving Asian immigrants.

**Results:**

Themes generated from constant comparative analysis suggest limited system-level preconditions and cross-organisational dynamics challenged integrated care. At the same time, changing organisational culture and practice, improving patient-provider and provider-provider communication, and increasing patient involvement enhanced clinical outcomes and facilitated successful implementation.

**Discussion and conclusions:**

Findings highlight the importance of patient involvement, peer services and interdisciplinary communication to successfully implement integrated care in the face of linguistic and operational challenges in settings serving multilingual and multicultural patients.

## Introduction

Individuals with serious mental illness (SMI; a diagnosable mental, behavioural, or emotional condition, excluding developmental and substance use disorders, of sufficient duration to meet diagnostic criteria specified within DSM-IV that has resulted in serious functional impairment) [1] on average die 25 years sooner than the general population [2]. Evidence shows many premature deaths could be attributed to preventable medical conditions (e.g. diabetes and cardiovascular diseases), a generally unhealthy lifestyle [3,4,5,6,7,8,9,10] and inadequate physical healthcare [11,12,13]. Physical comorbidities in individuals with serious mental illness often go undetected and untreated due to barriers to care [11,12,13]. Thus, it is important to address the service barriers in a fragmented healthcare system in which mental health has not incorporated physical care into their practice, despite the clear interrelationship between physical and mental health [3].

Primary care-behavioural health integration is defined as “the care that results from a practice team of primary care and behavioural health clinicians, working together with patients and families, using a systematic and cost-effective approach to provide patient-centred care for a defined population” [14]. There is a continuum of coordination and collaboration between primary care and behavioural health, ranging from coordinating services, co-locating services to integrating practices and building an interdisciplinary team of care [15]. Integrated care may be particularly effective for racial and ethnic minority populations with serious mental illness, given they face higher rates of chronic illnesses, lower rates of healthcare utilization and poorer quality of care than U.S. non-Hispanic White counterparts [7,16,17,18].

Several studies have demonstrated the potential effectiveness of primary care-behavioural health integration in increasing the use of physical healthcare services and enhancing quality of care as well as health outcomes among individuals with serious mental illness [19,20,21,22]. Small to medium effects were found in patients’ clinical symptoms, reported mental and physical quality of life, and social functioning [23]. Nonetheless, integration efforts remain difficult due to an insufficient understanding of the critical processes that bridge primary care and behavioural health services, and treatment elements that impact care [24,25,26]. A lack of diversity in patient samples further limited understanding of integrated care implementation in non-white populations [16,24,27]. As healthcare reform initiatives supporting integration continue to expand for individuals with serious mental illness, it is necessary to examine how primary care-behavioural health integration models can be implemented effectively, particularly in healthcare settings serving minority patients. The present study aimed to better understand the facilitators and barriers of integrated care in a multilingual behavioural health setting that primarily serves Asian immigrants in the United States.

## Theory and Methods

### Study setting

This study took place in a large non-profit community mental health clinic in a metropolitan area of California. The clinic provided comprehensive outpatient behavioural health services to more than 2,000 patients annually. Their patients were mainly low-income Asian immigrants of which 90% spoke a primary language other than English.

### Program description

In 2010, the clinic obtained government funding to initiate their Primary Care Integration program. The program collocated services in which primary care providers were embedded in the behavioural health setting [15]. The interdisciplinary team was comprised of thirty-six behavioural health providers (including one psychiatrist) from the community mental health clinic and three primary care providers from the adjacent federally qualified health centre that provides medical services to a large Asian population in the area. Patients, who were 18 or above, self-identified Asian Americans, and receiving mental healthcare through the clinic, were recruited by their care managers to enrol in this program.

Through this program, patients received primary care services and regular behavioural health care management in the same facility. Patients attended appointments with their assigned primary care physicians every three months. Bilingual/bicultural behavioural health care managers also joined primary care visits with patients to aid on language translation and follow-up on recommendations. The interdisciplinary team used an electronic health record system to coordinate appointments, obtain and share patients’ lab results, and to provide e-prescription. The psychiatrist, wellness coordinator, integrated care program administrators, care managers and primary care providers also held regular case conferences to discuss patients’ treatment goals and outcomes. As part of the program, integration of physical health care and wellness education was expected to be included in routine care. Patients might also be recommended to join various wellness activities based on their needs and availabilities. These wellness activities were typically staffed by a bilingual instructor and co-facilitated by a peer worker. Peer workers, who have lived experience of serious mental illness, were trained and supervised by behavioural health providers.

The primary care integration program was a four-year effort. During the final year, the authors collaborated with the staff in recruitment and data collection for this qualitative study to examine factors that supported (i.e. facilitators) or hindered (i.e. barriers) the implementation of the program.

### Theoretical framework and study design

The Health Behaviour Framework provides an overview of both contextual and individual factors that could influence patients’ health behaviours and outcomes [28]. It is commonly used in studies of health disparities among chronically ill populations. Based on this framework, the present study gathered perspectives from both providers/administrators and patients to capture factors at the system, organisation, provider and individual levels that could potentially influence the integrated care implementation. Components of the Health Behaviour Framework were used to generate interview questions. Questions for providers intended to elicit their perspectives on the implementation of the program, including the provider characteristics, practice patterns, and structural support and systemic barriers. Providers/administrators were interviewed individually as they came from different disciplines that might represent different perspectives. Questions designed for patients focused on understanding their access to, experiences and perceptions of care in the program. Focus group discussion was selected for patients as a format to encourage participation and in-depth discussion [29]. The study protocols were approved by DePaul University’s Institutional Review Board. Informed consent was obtained from each participant prior to participation.

### Sampling and recruitment

The authors emailed and recruited five providers/administrators (4 from behavioural health and 1 from primary care) for interviews. There were in total 36 behavioural health providers and 3 primary care providers in the program. These five providers/administrators were purposively selected for the study, based on their knowledge of and involvement in the planning and implementation of various aspects of the program, which allowed for rich and diverse perspectives [30]. Provider characteristics and roles were presented in Table 1.

**Table 1:** Provider Characteristics.

	*n* (%)	or mean (*SD*)

Gender		
Male	1	(20%)
Female	4	(80%)
Ethnicities		
Chinese	3	(60%)
Cambodian	1	(20%)
Vietnamese	1	(20%)
Years of professional service	10.6	(8.9)
Roles		
Primary care	1	Primary care physician
Behavioural health	1	Program director/clinical supervisor
	1	Program coordinator/clinical supervisor
	1	Wellness coordinator/clinician
	1	Data specialist/peer navigator

At the time of recruitment, 250 patients were enrolled in the program, 41 joined focus group discussions via convenience sampling. Recruitment flyers were distributed in individual and group meetings at the clinic. Patient characteristics were summarized in Table 2. All patients participated in the program were adults with monthly income less than $900 and were eligible for the state’s free or low-cost health coverage (i.e. Medi-Cal).

**Table 2:** Patient Characteristics.

	*n* (%)	or mean (*SD*)

Gender		
Male	12	(29.3%)
Female	27	(65.9%)
Age	52.1	(10.6)
Ethnicities		
Chinese	7	(17.1%)
Cambodian	14	(34.2%)
Filipino	1	(2.4%)
Korean	2	(4.9%)
Mien	7	(17.1%)
Vietnamese	6	(14.6%)
Thai	1	(2.4%)

*Note:* There were 2 missing cases for gender and age, and 3 missing cases for ethnicity.

### Data collection

Between March and May 2015, the authors conducted five one-hour semi-structured interviews with providers/administrators in English over the phone. In July 2015, the second author and bilingual staff conducted seven semi-structured focus group discussions with patients. Each one-hour group consisted of six to seven participants, with one bilingual staff as the interpreter. The groups were administered in languages concordant to participants. These languages were Cantonese, Vietnamese, Khmer, Mien and English. Participants were compensated with a $20 gift card. All interviews and discussions were audio-recorded.

### Data analysis

Bilingual/bicultural researchers transcribed the recordings into English. Constant comparison method was used [31], wherein analytic themes were generated independently by multiple trained coders, then refined and agreed upon in study team meetings via group discussion. Six trained coders independently read the transcripts and generated initial codes guided by the study aim. Following an iterative coding process [32], coders reviewed and compared the codes in team meetings to generate a codebook. Based on the same codebook, pairs of coders revisited the transcripts to understand the meanings and ideas of the codes. The authors, guided by the Health Behaviour Framework, met with the coders to discuss the abstract meanings, patterns and interconnections among the codes, as well as explored possible factors beyond the framework. After the team reached theoretical saturation at which no additional data could provide more information to the codes [33], no further focus groups and interviews were conducted. The team inductively generated themes and reached consensus on the meanings and importance of the themes. NVivo 10.0 [34] was used for the analytic process.

To enhance the credibility of the findings and ensure they are reflective of participants’ experiences, strategies to triangulate data sources and analysts were used [35]. First, multiple pairs of coders were employed to cross check data and achieve reliability. Coding reliability was assessed by comparing codes assigned to the same transcript by two different coders. Discrepancies in coding were discussed and clarified between the paired coders and in study team meetings. Second, we acknowledged potential differences in the information collected from focus groups and individual interviews, so similar and/or differing perspectives of patients and providers/administrators were taken into consideration in the process of coding and generating themes. Third, member checking was conducted through sharing the findings with six participants after data analyses. Participants agreed that the themes reflected their experiences and perspectives.

## Results

Current findings identified five themes that described the facilitators and barriers as crucial to successful implementation of integrated care. The themes were summarized with sample illustrative quotes in Table 3.

**Table 3:** Themes and Examples from Patients and Providers/administrators.

Themes	Sample quotes from patients	Sample quotes from providers/administrators

Limited preconditions at the system level	“I feel bad that inside the church when the clinicians always supplement the food they gave us and cooking by a lot. My clinician spends a lot her own personal money on it outside of it.”	“For some practitioners, it’s just been difficult, and that’s because not fully understanding the benefits of integration, benefits of understanding the whole person, and working as a team not working individually”, (behavioural health project coordinator)
Cross-organisation dynamics		“I have to say when I look back, I felt like we could do more team building and also role clarification…I hope there will be more time [for behavioural health and primary care providers] finding the common ground, finding the vision, and kind of scale back what they need to do in order to get to that point [integrate both sides’ services].” (behavioural health project coordinator)
Changes in organisational culture and system		“I have to say our care managers they are more willing [to] see the importance of bringing the client to their primary care providers appointments more than before.” (behavioural health project coordinator)
Improved patient-provider and provider- provider communication	“He had a gout, which he didn’t know what it was, and he was really thankful that his case manager was there to bring it up to have the discussion with the primary care doctor so he had a better understanding of his physical health.”	“I can recommend walking but who’s going to follow up? The case manager is taking them out for a walk…those are the kind of things I see…Or at least it would be reported back to me that they were doing this.” (primary care physician)
Increased patient involvement	“I volunteer in the exercise, yoga group, in Zumba whenever the instructors are out…1 just memorize the instructor’s combinations, and do the combinations more or less, and they’re just following along, the group members…. It makes me feel proud to be able to do anything good. I’m helping myself and I’m helping others at the same time.”	“The client is part of the team… [In] the past, even though the treatment person or the treatment team decide what’s best for the client. But now, we are incorporating the client to be a part of it. They have a sense of that… Because they know what’s been working for them, what they are willing to do.” (behavioural health clinician)

### Limited Preconditions at the System-level

From the outset, providers identified four gaps in the macro system of healthcare that challenge optimal implementation of integrated care. A foremost struggle was the lack of training in clinicians regarding primary care behavioural health integration. Other providers said that in an integrated care setting, additional training and time were required to modify interventions in ways that best engaged their patients and responded to patients’ needs.

Second, particularly in the context of serving a multilingual and diverse patient population, providers reported a constant struggle with inadequate resources to overcome cultural and linguistic barriers unique to the Asian immigrant population. Behavioural health providers said it was difficult to recruit multilingual clinicians who had knowledge, experience and willingness to work with non-English speaking populations with serious mental illness. The behavioural health clinician said she ended up being the instructor for most of the wellness activities because she was not able to find other instructors who felt comfortable to do so.

Third, behavioural health and primary care providers expressed concerns about the current reimbursement system that does not compensate for increased workload in integrated care setting. A behavioural health provider said:

“It has to be coming from the legislature all the you know, the state, and the government on the needs to have a different type reimbursement program for integrated care. Especially primary care, they are facing like you know from 10–15 minutes to half an hour visit. It is a lot of cost. And they don’t get compensated for that.”

The primary care physician stated her belief that the primary care clinic was losing money from their involvement in the integrated care program because it was difficult to obtain reimbursement for services rendered. Furthermore, other providers reported that the paucity of continuous funding hindered sustainability of the program. The program coordinator said many staff viewed the funding for the program as time-limited and therefore were not willing to make more permanent changes in support of integrated care efforts.

Despite the clinic could secure a space for providing colocation of services, patients and providers discussed the challenge of physical capacity, specifically access to facilities large enough to hold wellness groups as part of the integrated care program. A behavioural health provider explained that they had been using donated space at a church to conduct some wellness group activities. However, this facility was small and not always available. Whereas there was an intention to increase wellness programming, the existing physical barriers limited the variety of wellness activities held and the number of patients that could benefit from them.

### Cross-Organisation Dynamics

The primary care integration program was the first formal working relationship between the primary care and behavioural health providers serving mutual patients. Providers on both sides admitted that the differences between two organisations made it difficult to work together and put the integrated care model in practice. The primary care physician said:

“I feel like the first year it really did take a whole year to get started and to just kind of acclimate because everyone was so different in their perception and vision of what the primary care integration project should be.”

Providers raised challenges in creating a shared vision of integrated care, clarifying roles and responsibilities between behavioural health and primary care, and developing a mutual understanding of the administrative procedures (i.e. enrolment, scheduling, and billing) required for the integrated care program. Providers also reported working under constraints due to different protocols in two organisations. For example, the primary care physician mentioned they had to work around the issue of patient eligibility and billing for this program because primary care followed a different set of admission and insurance criteria required by federally qualified health centre. Providers expressed the importance of an interdisciplinary team to share vision, goals and priorities. They wished they had spent more time and opportunities on staff networking to build a cohesive team.

### Changes in Organisational Culture and System

Despite the myriad of organisational challenges, providers agreed that there were gradual changes that led to a new culture and operational system for integrated care. Behavioural health clinician reported seeing an increased willingness in care managers to work with medical providers. Primary care physician, on the other hand, took initiative to communicate more frequently with care managers and acknowledge the importance of behavioural health interventions. Providers recognized a change in mentality from “your patient, my patient” into “our patient” and viewed their patients as shared within the team. Providers also reported changes in the conventions of their jobs to improve service delivery. Care managers conducted more follow-ups with their patients on primary care providers’ recommendations; whereas primary care physicians spent more time with patients learning about their mental health conditions.

Behavioural health providers explained that these changes occurred in the context of continuous access, exposure and education on each other’s work. The behavioural health clinician and program coordinator said they took time to educate the care managers and primary care physicians about holistic wellness and invite them to see the different parts of the program and how they could apply in their practice. The program coordinator said:

“I really think that the immediate providers see the benefit and really they have different takes towards the care, the approach.”

### Improved Patient-Provider and Provider-Provider Communication

Both behavioural health and primary care providers reported using formal and informal communication channels to exchange information and provide structures for accountability, differentiation and coordination of care. The psychiatrist, primary care providers, care managers and other behavioural health staff held regular case conferences to discuss patients’ treatment goals and progress. A few providers mentioned using daily informal check-ins amongst themselves to avoid patients falling through the cracks of the system and ensure services from different parties were provided to meet the patients’ needs. Information exchange allowed providers to integrate their services for patients and be better informed about the feedback of their recommendations. Additionally, some patients saw their linguistic barriers and discomfort with primary care providers in discussing mental health issues being removed by having linguistically matched care managers at primary care appointments, thus improving patient-provider understanding.

Providers reported using an information sharing system to facilitate the integration between behavioural health and primary care. In the second year of the program, behavioural health providers obtained funding to develop their electronic health record system. The program coordinator explained that they used the system in three ways: (1) psychiatrists made e-prescriptions and checked for potential interactions among patient’s different medications; (2) behavioural health providers obtained lab results to keep track of patients’ health and progress; (3) primary care providers had access to the system to schedule patients’ appointments. Despite some legal and technical barriers, providers valued the electronic health record system as it allowed immediate access to information at anytime from anywhere, and to coordinate appointments or treatment efforts, which was “especially important in this hard-to-reach population when no-shows are common”.

### Increased Patient Involvement

All providers recognized the importance of involving patients’ knowledge, strengths, needs, perspectives and participation in supporting the implementation of integrated care and enhancing patients’ clinical outcomes. They saw patients as “navigator[s] of their own recovery” and valued patients’ awareness and abilities to manage their health. Providers reported more discussion with patients regarding their vital signs and health data. Family members were also invited to wellness activities with patients in order to reinforce health and wellness management at home.

Peer involvement was another initiative in the integrated care program. Several patients shared their experiences in volunteering and assisting certain parts of the integrated care program, such as recruiting patients, co-facilitating wellness activities, and providing support in language translation. These patients reported their experiences of leadership motivated them and their peers. Behavioural health providers said they created positions for peer leaders and recruited patients who had received training from the clinic. Parts of the training focused on culturally responsive and therapeutic engagement, and being sensitive to legal as well as ethical issues. The clinic encouraged peer involvement by providing supervision and acknowledging their services with stipend or gift cards. Providers described peer leaders as “valuable workforce member[s]”, since peer leaders provided additional resources and unique expertise to support integrated care implementation under an already taxed environment.

## Discussion

The study provides insights into the implementation process of primary care-behavioral health integration and highlighted facilitators and barriers of employing a co-located care model in settings serving multilingual and multicultural patients. Our findings suggested that limited system-level preconditions and cross-organisational dynamics hindered integrated care implementation. At the same time, improvements in organizational culture and system (i.e. providers’ mentality, job conventions), improved patient-provider and provider-provider communication, and increased patient involvement helped facilitate the implementation process. Based on these findings, we provided recommendations for future implementation. (See Table 4).

**Table 4:** Recommendations for future implementation of integrated care.

Major activities in this primary care integration program		Future recommendations

1.	Co-locate services Primary care providers embedded in behavioural health setting	Provide training to future providers and staff on integrated care prior to implementation Consider organisational culture and top-down involvement in planning for integrated care
2.	Create an interdisciplinary team Recruit primary care physicians, psychiatrists, bilingual care managers, behavioural health clinicians and administrative staff	Invest time and resources in team building and defining shared vision of integration Develop shared protocols and clarify responsibilities with expected outcomes
3.	Develop channels for greater interprofessional communication Regular case conferences Informal check-ins and referral procedures Bilingual behavioural care managers attend primary care appointments with patients	Evaluate reimbursement, billing and funding systems Management commitment to protect time and resources for integrated care activities and training Develop financial and technical capacity in using and maintaining an information sharing system
4.	Set up an electronic health record system For e-prescription, obtaining lab results and coordinating patients’ appointments	Allow different existing electronic health record systems to be compatible with one another Develop culturally responsive treatment plans and wellness activities for patients and their families
5.	Engage patients and family in treatment and wellness activities	Share decision making process with patients through involving bilingual care managers
6.	Enlist peers to be part of the workforce and provide training and leadership opportunities	Create positions, define roles and provide training, incentives and supervision for peer leaders

At the system level, workforce challenges and payment structures for integrated healthcare services imposed constraints on optimizing integration of care. Whereas other studies reported the need to train clinicians with knowledge, attitude and skills to deliver integrated care services [26,36], our findings also added the importance of having multilingual, culturally sensitive clinicians. To develop integrated care interventions that can meet the unique needs of ethnic minority patients, more resources should be invested in recruiting and training both diverse providers [37]. Training and workforce development should begin early in medical and mental health education to effectively equip future providers with the skills and mindsets necessary for an integrated care approach. Cross-training activities between primary care and behavioural health are suggested to enhance mutual understanding of the roles and duties. Training on cultural-specific conceptions of health, wellness, connection between the mind and body, and stigma about physical and mental illness would enhance quality of integrated care for culturally diverse individuals.

Removing system-level financial barriers is another prerequisite to support integrated care. Our findings suggest the need to modify existing funding models to allow for compensating the unique services (e.g. drop-in appointments, warm hand-offs where one provider physically guided patients to another provider) used in many integrated care settings. Streamlining funding structures and providing continuous funds are required to sustain integration [38]. For example, the use of electronic health record system facilitated providers to share data and coordinate treatment efforts. However, building an information system that allows collection and maintenance of information from multiple agencies require adequate IT infrastructure and financial support [38]. Currently, behavioural health providers are not reimbursed for implementing electronic health record systems through HiTech incentives from the Federal government [39]. Technical and legal challenges further impeded the sharing of information in the system. It is noted that current providers in behavioural health clinics and primary care settings used different electronic health record systems that are incompatible with one another, thus making communication and exchange of information across systems difficult. Extensive training was also needed for staff in the current study to become familiar with using these information sharing systems. Policymakers and funders must take into account the additional burdens on staffing and other resources associated with caring from multilingual, multicultural patients, as suggested elsewhere [40].

At the organisational level, the findings highlight the importance of considering organisational culture and top-down involvement as they shape the ability to move beyond minimal collaboration to closer integration of care [41]. Organisational differences and dynamics between behavioural health and primary care made early transitions difficult and less satisfactory, especially when there was minimal organisational and administrative support. Establishing a shared vision and understanding of integrated care is required for successful collaboration between organisations. The literature suggested that within interdisciplinary teams, it is also critical to have clarity about the specific aspects of care for which individuals in the team are responsible and accountable, supported by effective communication between team members [38]. Shared protocols, joint action plans and decision-making tools could be helpful in setting responsibilities with expected outcomes for providers. All providers and staff need time for training and to collaborate on patient care, which can be difficult in taxed clinical settings. Management commitment to protect time and resources for such activities are needed.

At the providers’ level, the formation of an interdisciplinary team and effective communication between primary care and behavioural health providers have long been acknowledged as necessary to improve the physical health of individuals with serious mental illness. Our providers adopted various inter-professional communication strategies, such as regular case conferences and informal referral procedures, that promoted team effectiveness [24]. Operational changes such as modifying workflow and process of care, using data to track outcomes and evaluate improvements, providing primary care providers with access to shared information systems, and making changes related to leadership and practice culture are required [42]. Current findings reported changes in providers’ perceived ownership and responsibility of patients as shared within the team. Changes to traditional job conventions, such as primary care providers extending consultation time and care managers attending primary care providers’ appointments with patients, were also noted as critical to implementation success. These changes require willing, interested, committed and passionate staff, plus investment and commitment to change from all stakeholders [41,43].

At the individual level, patient involvement is underscored as an active intervention ingredient by our providers and patients. They reported that communicating to patients about their health data and involving them in decision making, service delivery and informal peer support increases patients’ self-efficacy, sense of responsibility, and motivation for individual behavioural change. Shared decision making has been frequently advocated in recovery-oriented behavioural health care and medication management, but seldom used in behavioural interventions for persons with serious mental illness [44]. The experiential expertise unique to patients also improved treatment effectiveness and patients’ satisfaction with care [45,46,47]. Furthermore, peer workers and support leaders play a very critical role for integrated care in multilingual settings given their linguistic and cultural capacity along with their experiential knowledge. Our study showed peer workers serve as co-facilitators, navigators and language interpreters. By helping to serve as an interface between patients and the treatment team, peer leaders contribute to patients’ greater treatment engagement and improved health outcomes [48]. Peer leaders also help to supplement the gaps in services where there is a lack of cultural competency and resources.

Integrated care programs are increasingly incorporating peer support leaders into delivery of services; yet the process of implementing and ways of supporting peer support leaders in integrated health care settings are still unclear [49]. Our study showed successful peer involvement requires organisational commitment, training, supervision, incentives and providers’ support and trust. To expand peer services in integrated care for multilingual patients, continuous investment in training and incentives from clinics are needed for peer leaders who help address cultural and linguistic challenges, for peers to mentor one another, and for providers to work effectively with and guide peer leaders [37].

Our study had several limitations. Convenience sampling was used for recruitment in this traditionally hard-to-reach population, and possible selection biases were noted. Most patients in the focus groups received both integrated care services and wellness activities versus those who only received integrated care services, thus the focus groups were not expected to be representative of all patients. The sample of our providers, who came overwhelmingly from behavioural health versus primary care, might not be representative of all providers; however, this was reflective of the integrated care team that was mainly composed of bilingual behavioural health clinicians. Given our study was conducted in one ethnic-specific community mental health clinic in California, it is acknowledged that the transferability of our findings is limited to Asian American adult immigrants with serious mental illness who were receiving care in a metropolitan area. Asian immigrants are a heterogeneous population and current findings should be interpreted with caution when applying to a particular Asian ethnic group. Multilingual staff were employed as interpreters in focus group discussions to facilitate understanding between researchers and patients. The risks of misinterpretation and social desirability bias were noted. Nevertheless, our findings provide important directions to implement integrated care in high disparity, ethnic-specific and multilingual populations.

## Conclusion

Addressing health disparity requires prevention and care for comorbid medical conditions. Promotion of healthy behaviours, early diagnosis and coordinated management, and integrated care between the mental health and medical systems are necessary. For linguistically and culturally diverse populations with serious mental illness, future integrated care efforts should focus on incorporating ways to engage patients in treatment, decision-making and service delivery processes, remove structural barriers for systems change, create opportunities to promote inter-professional team building and functioning, better allocate resources, and increase funding to build capacity for long-term sustainability [24]. Present findings help to inform future integrated care program development and implementation by pointing to active ingredients that may facilitate individual and organisational change and barriers that compromise implementation. They also assist organisational leaders and policymakers to make practical decisions regarding clinical, operational and financial needs. Integration of primary care and behavioural health is important to reducing disparities among those with serious mental illness, but such care is not “one size fits all” [24]. Approaches to integration should be responsive to the needs and context of the community. Therefore, providing empirical evidence to evaluate what works and what does not work for linguistically and culturally diverse groups is important.

## Additional File

The additional file for this article can be found as follows:

10.5334/ijic.3719.s1 Table S1 Themes and Examples from Patients and Providers.
