# Supplementary material for: A Qualitative Study on Primary Care Integration into an Asian Immigrant-specific Behavioural Health Setting in the United States
Source: Int J Integr Care. 2018 Jul 3;18(3):2. doi: 10.5334/ijic.3719 (PMC6133215; doi:10.5334/ijic.3719)
Supplement: Table S1 — Themes and Examples from Patients and Providers. [file ijic-18-3-3719-s1.pdf]

Table 1

Themes and Examples from Patients and Providers

Themes	Sample quotes from patients	Sample quotes from providers/staff
Patient as part of the treatment and workforce team	"We come here to learn how to help yourself and myself. And to share with other people." "I volunteer in the exercise, yoga group, in Zumba whenever the instructors are out…I just memorize the instructor's combinations, and do the combinations more or less, and they're just following along, the group members…. It makes me feel proud to be able to do anything good. I'm helping myself and I'm helping others at the same time."	"The client is part of the team…[In] the past, even though the treatment person or the treatment team decide what's best for the client. But now, we are incorporating the client to be a part of it. They have a sense of that… Because they know what's been working for them, what they are willing to do." (behavioural health clinician)
Improved patient-provider and provider-provider communication	"He had a gout, which he didn't know what it was, and he was really thankful that his case manager was there to bring it up to have the discussion with the primary care doctor so he had a better understanding of his physical health."	"I can recommend walking but who's going to follow up? The case manager is taking them out for a walk…those are the kind of things I see…Or at least it would be reported back to me that they were doing this." (primary care providers)
Cross-organization		"If I look back, I hope there will be

dynamics		more time finding the common ground, finding the vision, and kind of scale back what they need to do in order to get to that point." (behavioural health project coordinator)
Changes in organizational culture and system		"I have to say our care managers they are more willing [to] see the importance of bringing the client to their primary care providers appointments more than before." (behavioural health project coordinator)
Operational and financial barriers	"I feel bad that inside the church when the clinicians always supplement the food they gave us and cooking by a lot. My clinician spends a lot her own personal money on it outside of it."	"We have clinicians… who they never had any experiences working with our diverse population, specifically with an API [Asian and Pacific Islanders] serious mental illness population. It's very new, something they don't really receive in school. (behavioural health clinician)
